# The Facts of Syphilis on the Stage: Plays by an English Doctor and a French Poet

**Published:** 1914-05-16

**Authors:** 


### THE FACTS OF SYPHILIS ON THE STAGE.

### Plays by an English Doctor and a French Poet.

One of the immediate results brought about by the formation of a Royal Commission on Venereal Diseases, of which the Commission itself is a symptom, has been an active agitation for educating the general public in the facts of syphilis. The witnesses before the Commission have been practically unanimous that this education must somehow be accomplished, but we are almost as far off as ever from seeing it carried out owing to the opposition which so far has greeted all attempts made in this direction. It is idle to pretend that this opposition is mere prejudice and prudery. It is not. The truth of this is apparent from the violent scenes which recently occurred in a provincial school where a certain teacher, apparently quite destitute of discretion or responsibility, took upon herself to give instruction in the facts of sex to her class of girls, whose parents made the country ring with their protests. Clearly, if there is to be education in the subject, as all responsible persons acquainted with the facts and realising the dangers of ignorance are agreed, it must be given in the proper way and at the proper time by instructors carefully chosen for their capacity. But what is the proper way, and when is the right time, and who is the proper instructor? Sir Victor Horsley pointed out the direction which an answer to these vital questions must take when, in the course of his recent evidence before the Royal Commission, he advocated class-room instruction in the reproductive systems of plants and animals, and emphasised the desirability of restricting information on reproductivity in man to private individual tuition. If the mothers and parents generally could be trusted to carry out their duties in these respects all would be well; but the fact is that they notoriously cannot be so trusted, and therefore have only themselves to blame for such irresponsible action on the part of individual teachers as that already referred to.

The fact which we believe to be important is that the general public, rather than the individual young person, ought to be the first object of educational propaganda, because once the general public has become as accustomed to discuss the ordinary facts of venereal disease in public as it is to discuss and listen to discussions on the facts of politics, sport, or art, then its individual units will be equipped to some extent to pass their knowledge on to their families without embarrassment, and to *require* such instruction for their children from properly equipped teachers as they do now in other non-tabooed subjects like music or French.

For this reason we turn with considerable interest to two plays* which have been published by Mr. Fifield, the one, entitled " Philip's Wife," by Mr. Frank G. Layton, M.R.C.S., already known to our readers as a contributor to The Hospital over the signature " Stephen Andrew," and to the general public as the author of a very capable novel on provincial general practice entitled " Dr. Gray "; and the other,† entitled " Damaged Goods " (" Les Avariés "), by M. Eugène Brieux, the famous

* *Philip's Wife.* A Play by Frank G. Layton, M.R.C.S. (A. C. Fifield, 13 Clifford's Inn. 1s. net.)

† *Damaged Goods.* Translated from the French of Brieux by John Pollock, with a Foreword by Mrs. Bernard Shaw. (Fifield. 1s. net.)

French dramatist and Academician. Both works are issued at 1s. Taking first "Damaged Goods," as the more famous work, we may note in passing that it has been translated by Mr. John Pollock, and is preceded by a Foreword from the pen of Mrs. Shaw, which is followed by an extract from the preface to "Three Plays by Brieux," which her husband contributed to that volume on its publication a year or two ago.

If we are right in our contention that the education of the public in the facts of venereal disease should begin by relating them to men and women in the mass, then two things become evident. First, that the theatre is an admirable platform from which to start; and second, that the method of instruction must be made arresting to simple people, who will expect some other bait for their attention than the mere relation of the truth. The theatre, indeed, has a double claim to the honour of a first place in this educational campaign. First because the sex-attraction, which so often results in venereal disease, has always been regarded as its peculiar perquisite, and is, in fact, exploited there to the utmost limit of human ingenuity without let or hindrance; so that if the theatre be allowed to present one side so arrestingly, it must also, in the interests of public health and public morality, be allowed to present the other. And secondly, that if the disastrous effects of venereal disease have become so widespread as to earn the bishops' title of "the social evil," it is clear that the platform chosen for exposing it must be one of equal social appeal. The theatre to-day is the most public and social of all institutions, therefore clearly the theatre is a very good place in which to discuss and explain the one evil to which the adjective "social" is applied.

Having reached this inevitable conclusion, let us pass on to consider the manner of presentation, which, as we have seen, is as vital as the presentation itself. Before the curtain rises on "Damaged Goods" M. Brieux instructs the manager of the theatre to come before the curtain and to say: "Ladies and gentlemen, I beg leave to inform you, on behalf of the author and of the management, that the object of this play is a study of the disease of syphilis in its bearing on marriage. It contains no scene to provoke scandal or arouse disgust, nor is there in it any obscene word; and it may be witnessed by everyone, unless we must believe that folly and ignorance are necessary conditions of female virtue."

How far does the play bear out the claim made for it? The best answer to this question is for readers to study the play themselves. But to those who have not done so we may say that there is nothing in it to harrow our nerves, except the ignorance of the victims; there is nothing to arouse our disgust, except the way in which medical science is blocked at every point; and there is nothing to make our daughters blush, except the exposure of the risk which parents allow them to run without warning, care, or sense of responsibility. If the play is stern, it is stern as facts are stern by their simplicity and rock-like firmness; if it is arresting in the highest degree, it is because the struggle—that between the doctor on the one hand and the ignorance, fear, and cowardly refusal to think of anything beyond immediate personal gratification on the part of the infected man and his relations on the other—appeals to our consciences as vital, tragic, and personal to ourselves in the highest sense. These qualities apply in different degrees to both the plays before us. But whereas Brieux never lets the tension slacken, nor allows our attention to flag, but holds us fascinated to the end, Mr. Frank Layton is not quite so sure a technician. All that the importance of his subject can give him he takes, and allows us to attend to without any adventitious aids to heighten the effect—he is almost as quiet as St. John Hankin in his manner; but he lacks, at present, the grip of the master dramatist. It is not wasting time to pause a moment and resolve this defect into its elements. What is, to take an illustration, the thing which holds our attention unfalteringly throughout the dialogue of "Damaged Goods," which we miss, in places, in the dialogue of "Philip's Wife"? Both appear equally natural and true to conversation as we know it. But we feel a difference to be there. It is to be found in the whole difference between poetic and prose drama. Bunyan was a poet, though he never wrote a line of verse except a bad one; Brieux, though he has never to our knowledge published poems, is a poet of the same order as Bunyan. Now the difference between poetic and prose drama has been put very clearly and truly by Mr. Arthur Symons in these words (which we quote from memory): "In prose drama the characters say exactly what they would say in real life. In poetic drama they say the most self-revealing thing that they could say." That is why "Damaged Goods" is a finer play than "Philip's Wife." But do not let Mr. Layton fancy any dispraise lurking in this explanation. The fact may do the cause that he has at heart—and he says in his preface that his aim is "to help to kill the superstition that women should be kept in ignorance of certain subjects which affect them vitally"—no harm. For the poetic method, while it attracts the clearest heads and the most vivid imaginations, affects those without these qualities, like the general public whom we need to reach, in the same manner as a great fire: it does not arouse them so much as frighten them, and when they are frightened they run away, and we do not want them to run away from any theatre when either of these plays is being performed. The fact is, of course, that whatever inspiration or alarm these plays will give must at present, we fear, be confined to the reading public. The Censor stands in the way, and as under our Constitution the Reader of Plays happens to be a court official, from training and prejudice, almost as much as from the nature of his office, he combines the worst as well as the best elements in conservatism. Now, as the best element in conservatism is sanity, or caution, so the worst is simple obstructiveness. An educated public opinion would soon remedy this, however, and that is why we welcome a doctor-dramatist to the rescue.

